# Editorial: Cross Talk Between the Immune System and Metabolism

**DOI:** 10.3389/fendo.2020.00588

**Published:** 2020-09-24

**Authors:** Jie Chen, Wen Kong, Jixin Zhong

**Affiliations:** ^1^Jiangxi Provincial People's Hospital Affiliated to Nanchang University, Nanchang, China; ^2^Department of Endocrinology, Wuhan Union Hospital, Tongji Medical College, Huazhong University of Science and Technology, Wuhan, China; ^3^Department of Rheumatology and Immunology, Tongji Hospital, Tongji Medical College, Huazhong University of Science and Technology, Wuhan, China; ^4^Department of Medicine, Case Western Reserve University, Cleveland, OH, United States

**Keywords:** immune system, metabolism, metabolic disease, chronic inflammation, metabolic dysregulation

Metabolic dysregulation leads to a number of diseases including diabetes, obesity, hypertension, gout and rheumatoid arthritis (RA). These diseases have been associated with chronic inflammatory processes. To date, increasing evidence indicates that immune system plays an important role in the process of metabolic diseases. For example, the activation of type 1 immunity was identified in obesity-associated metabolic dysfunction (1). Metabolism has been shown to play a critical role in RA, a chronic autoimmune disease with involvement of a series of pro-inflammatory and immune-regulatory cytokines and mediators (2, 3). Innate immunity is also of key importance in the pathogenesis of gout (4). Although the association between the immune system and metabolic diseases have been well-recognized, the understanding of immune-related mechanisms of these diseases are not fully understood. This special issue exhibits a number of original research articles and review papers on the topic of cross talk between the immune system and metabolism.

As a central immune organ, the thymus provides a place for naive T cell differentiation, development and maturation. With thymic senescence, the epithelial network shrinks and is replaced by adipose tissue, leading to a decline of its immune function (5). Meanwhile, beige adipose tissue plays a key role in metabolism. By investigating the beige-specific and beige-indicative markers and metabolic profile (OCR/ECAR ratio), Banfai et al. reported in this issue that thymic adipose tissue emerging with senescence was actually beige adipose tissue, which builds a bridge between immune organ and metabolic tissue. T cells develop within the thymus and play a central role in adaptive immunity. Sirtuins are nicotine adenine dinucleotide (NAD+)-dependent enzymes involved in the cell metabolism (6). A review by Jonathan L et al. summarized some recent progresses in the role of sirtuins in regulating adaptive immunity (Warren and MacIver).

Alarmins play vital roles in innate and adaptive immune responses and participate in a wide range of pathophysiological processes such as inflammation and oncogenesis (7). Guo et al. investigated how sodium butyrate exerts its anti-inflammatory activity by inhibiting an “alarmin,” HMGB1, and thus exhibits an anti-diabetic effect in type 1 diabetes. Shang et al. examined the role of another alarmin, IL-33, in an animal model of human gout, MSU-induced inflammation. A review by Tu and Yang summarizes the potential mechanisms of IL-33/ST2 axis in the metabolic disorders.

Hormones have regulatory effects on immunologic processes. In this special issue, a research by Porchas-Quijada et al. evaluated the relationship of anti-ghrelin autoantibodies with clinical, body-composition, and metabolic parameters in RA patients. Their findings support the previously reported functions of these natural autoantibodies as carriers and modulators of the stability and physiological function of natural hormones Porchas-Quijada et al.. In addition, a mini review by Montesinos and Pellizas summarized the cellular and molecular mechanisms involved in thyroid hormones effects on innate immunity. In recent years, studies into the microbiomes have revealed their close relationship with human diseases (8). A study by Ning et al. investigated the alterations of urinary microbiome in gout patients, indicating the new prospects for microbiome in the diagnosis and treatment of gout.

Metabolic disorder can lead to serious complications. For instance, diabetic cardiomyopathy and encephalopathy are common severe complications of diabetes that cause mortality and morbidity in diabetic patients. Using mouse models of diabetes, Bhusal et al. investigated the role of LCN2 in the pathogenesis of diabetic encephalopathy, which help explain the pathogenic mechanisms that cause this complication. Ying et al. found that Phloretin prevented diabetic cardiomyopathy, possibly by suppressing the interaction between Nrf2 and Keap 1. Their work indicates a suppressive effect of Phloretin in high glucose-induced injury of cardiomyocytes. Intensive anti-diabetic therapy in diabetic patients may cause hypoglycemia, which has been found to be associated with an increased risk for adverse cardiovascular outcomes and all-cause mortality (9). In this special issue, Wei et al. investigated the association between hypoglycemia as assessed by continuous glucose monitoring and the major adverse cardiovascular events or all-cause mortality.

Collectively, the original research and review articles in this special issue cover a series of important aspects in the field of interaction between the immune system and metabolism, which may provide new insights into the diagnosis and treatment of metabolic diseases and their complications.

## Author Contributions

All authors listed have made a substantial, direct and intellectual contribution to the work, and approved it for publication.

## Conflict of Interest

The authors declare that the research was conducted in the absence of any commercial or financial relationships that could be construed as a potential conflict of interest.
